# Macroscale evolutionary patterns of flight muscle dimorphism in the carrion beetle *Necrophila japonica*

**DOI:** 10.1002/ece3.15

**Published:** 2011-09

**Authors:** Hiroshi Ikeda, Teiji Sota

**Affiliations:** 1 Department of Forest Entomology, Forestry and Forest Products Research Institute1 Matsunosato Tsukuba, Ibaraki 305-8687, Japan; 2 Forestry Sciences Laboratory, USDA Forest Service320 Green Street, Athens, Georgia, 30602-2044; 3 Department of Zoology, Graduate School of Science, Kyoto UniversitySakyo, Kyoto 606-8502, Japan

**Keywords:** COI, flight ability, genetic diversity, phylogeography, Silphidae

## Abstract

Some insect species exhibit polymorphisms in flight muscles or wings, which provide opportunities for studying the factors that drive dispersal polymorphisms and the evolution of flightlessness in insects. We investigated the macroscale evolutionary pattern of flightlessness in the widespread Japanese beetle *Necrophila japonica* (Coleoptera: Silphidae), which exhibits flight muscle dimorphisms using phylogeographic approaches. *N. japonica* lives in both stable and unstable habitats, and the flight muscle dimorphisms may have been maintained through the use of these diverse habitats. We studied the distribution pattern of the proportion of individuals lacking flight muscles in relation to the genetic differentiation among geographic populations using an 842-base pair sequence of the COI-II gene. Both flight-capable and flightless individuals occurred over the distribution area, and the flight muscle condition showed no significant phylogeographic pattern. Several populations comprised flight-capable individuals only, whereas few comprised flightless ones only. Demographic expansion was suggested for major clades of COI-II haplotypes, and the genetic differentiation showed an isolation-by-distance pattern among the populations in Japan. The proportion of flightless individuals was higher in a population with a higher annual mean temperature and with higher genetic diversity among individuals. These results indicate that geographic expansion occurred recently while flight muscle dimorphisms have been maintained, that flight-capable individuals have colonized cooler (peripheral) habitats, and that flightlessness has increased in long-persisting populations as suggested by high genetic diversity.

## Introduction

Insects acquired flight capabilities approximately 400 million years ago (Engel and Grimaldi 2004). The ability to fly has enabled dispersal to various habitats and regions (Roff 1986, 1991). However, many insect species from diverse lineages have lost flight capabilities through the reduction, degeneration, or loss of flight muscles or wings (Roff 1990, 1994; Wagner and Liebherr 1992) because these flight apparatuses are costly to produce and maintain (Roff 1991; Lövei and Sunderland 1996; Zera and Denno 1997). In general, flightlessness in insects can develop upon establishment in isolated stable habitats with little spatiotemporal variability and high predictability (Harrison 1980; Roff 1990; Wagner and Liebherr 1992).

Species of several insect orders exhibit variations in flight ability due to polymorphisms in flight muscles or wings. These may be caused by genetic variations (polymorphisms) and/or by phenotypic plasticity (Zera and Denno 1997). In particular, wing dimorphisms in holometabolous and hemimetabolous insects are typically controlled by a single locus and polygenes, respectively (Roff and Fairbairn 2007). Although the relative importance of genetic variation and plasticity varies with the organ, species, and type of adaptation, it is likely that variations in flight ability due to both mechanisms are maintained by heterogeneous conditions in space and time based on the energetic trade-off between dispersal power and reproductive capacity (Roff and Fiarbairn 1991; Zera and Denno 1997; Roff et al. 1999; Fox and Czesak 2000; Jonsson 2003). Phylogeographic studies of species exhibiting variations in flight ability are important for understanding the factors affecting the evolution of flightlessness in insects.

**Figure 1:**
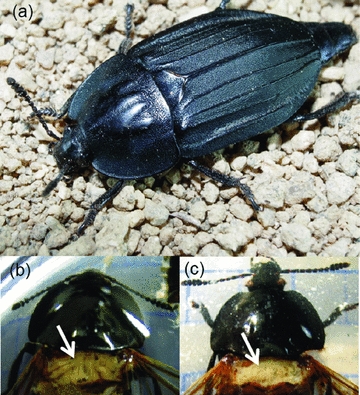
(a) A male beetle of *Necrophila japonica*. (b), (c) Specimens of *N. japonica* dissected to show the presence (b) and absence (c) of flight muscles. Arrows indicate flight muscles in (b) and non-muscle tissues in (c). Photo credits: T. Sota (a) and H. Ikeda (b, c).

In carrion beetles, flight ability has been lost in two lineages of the subfamily Silphinae (Coleoptera: Silphidae) (Ikeda et al. 2008b). One species, *Necrophila japonica*, which inhabits the Japanese islands, exhibits flight muscle dimorphisms in both sexes (Fig. 1; Ikeda et al. 2007). Because individuals of both flight muscle types possess fully developed wings (Ikeda et al. 2007), flightlessness in this species results entirely from the lack of developed flight muscles. The genetic basis of flight muscle dimorphisms is unknown in *N. japonica*. However, the flight muscle dimorphism in Coleoptera may share a common genetic system with the wing dimorphism of holometabolous insects; that is, a single Mendelian locus with flightlessness being dominant (Roff and Fairbairn 2007). Indeed, this system has been confirmed in a scarabaeid beetle (Tada et al. 1994). We have not found any seasonal change in the proportion of the individuals with flight muscles in *N. japonica* (H. Ikeda unpubl. data), and individuals with flight muscles lay many eggs (Ikeda et al. 2008). Thus, histolysis of flight muscles is not likely to occur in this species. *N. japonica* inhabits various types of habitats ranging from disturbed riparian zones to relatively stable forests and is often numerically dominant in the ground invertebrate community (Nagano and Suzuki 2003; Ikeda et al. 2008a, 2010; Shibuya et al. 2008). Flight muscle type does not correlate with diet or reproductive investment (Ikeda et al. 2007, 2008b). Therefore, we have not found any clear relationship of the flight muscle dimorphism with divergent ecological niches or the trade-off between dispersal and reproduction. Nonetheless, individuals with flight muscles would have more ability to colonize newly established or unstable habitats than flightless ones (Zera and Denno 1997).

We investigated the macroscale evolutionary process of flight loss in *N. japonica* by examining the relationships between the geographic pattern of flight muscle condition, genetic diversity within and among populations, and the phylogeographic pattern of this species in Japan. Since flight-capable individuals colonize peripheral or new habitats more frequently than flightless individuals (e.g., den Boer 1970; Liebherr and Hajek 1986), and newly established populations are likely to possess low genetic diversity due to bottlenecks, we hypothesize that genetic diversity within a population is negatively correlated with the proportion of flight-capable individuals in the population. Further, since peripheral or historically new habitats (e.g., those that became favorable in the post glacial period) exhibit particular climatic conditions (e.g., cool temperatures), we also expect that the proportion of flight-capable individuals is correlated with climatic variables such as temperature. The proportion of flightless individuals may be higher in relatively long-lasting populations in stable habitats and/or areas that did not experience the impacts of the last glacial period.

## Materials and Methods

We collected adult and larval samples of *N. japonica* from a wide range of populations across the Japanese archipelago, covering the species’ distribution area (Kurosawa 1985). We dissected adult beetles to determine the presence or absence of flight muscles. The samples used for genetic analysis as well as additional samples collected during field studies were dissected. We were not able to determine the flight muscle status of larvae and some adult beetles of which tissues were rotten due to poor fixation. Samples examined by Ikeda et al. (2007, 2008b) were also included in the analyses. To examine differences in the proportion of beetles with flight muscles between sexes, we conducted a Fisher's exact test using R 2.11.1 (R Development Core Team 2010).

We used an 842-base pair sequence of the mitochondrial COI-II gene, which corresponded to positions 2221–3059 of the *Drosophila yakuba* mitochondrial DNA (Clary and Wolstenholme 1985), for the genetic analyses. The methods for DNA extraction, polymerase chain reaction (PCR), and sequencing were the same as those described by Ikeda et al. (2009). The following PCR primers were used to amplify and sequence the COI-II region: COS2183N (forward), 5′-CAR CAY YTA TTY TGR TTY TTY GG-3′ (Sota and Hayashi 2004); C1-J-2195 (forward), 5′-TTG ATT TTT TGG TCA TCC AGA AGT-3′ (Simon et al. 1994); 1J2441 (forward), 5′-CCA ACA GGA ATT AAA ATT TTT AGA TGA TTA GC-3′ (Simon et al. 1994); COA3107S (reverse), 5′-TCY ATY ARA GGK GAR GCW CTR TCT TG-3′ (Ikeda et al. 2009); COA3186E (reverse), 5′-ATT AAG TAT CCG ACT AAA ACA G-3′ (present study); COA3374 (reverse), 5′-TAT CAT TGA TGX CCA ATA GTT TT-3′ (Ikeda et al. 2009); 2N3661 (reverse), 5′-CCA CAA ATT TCT GAA CAT TGA CCA-3′ (Simon et al. 1994). Data generated by Ikeda et al. (2009) were also included in this study. All haplotype sequences were deposited in GenBank (accession numbers: AB376112, AB376114, and AB606505–AB606596).

We calculated nucleotide and haplotype diversities and conducted analyses of molecular variance using ARLEQUIN version 3.11 (Excoffier et al. 2005). We then examined isolation by geographic distance using Nei's genetic distance *D_A_* and ln-transformed geographic distance as variables. To assess the relationship between geographic and genetic distance, we conducted a Mantel test with 10,000 permutations using ARLEQUIN version 3.11. A statistical parsimony network of the COI-II gene was constructed using the program TCS version 1.21 (Clement et al. 2000). Loops in the networks were resolved according to the three criteria described by Pfenninger and Posada (2002). To examine demographic expansion, we performed tests of the stepwise expansion hypothesis by calculating the sum of square deviations (SSDs) between the observed and expected mismatches (Schneider and Excoffier 1999) and the population equilibrium hypothesis by Fu's *Fs* (Fu 1997) for constructed clades using ARLEQUIN version 3.11. A significantly large SSD indicates departure from demographic expansion, and a significantly large negative *Fs* indicates demographic expansion.

To elucidate the phylogeographic pattern of flight loss, we examined the differences in the ratio of flight-capable individuals between clades using a Fisher's exact test with R 2.11.1. We then simultaneously examined the effects of genetic diversity and climatic conditions on the proportion of beetles with flight muscles within populations using a generalized linear model (GLM) analysis. Note that, although we included the intrapopulation genetic diversity as an explanatory variable in the GLM analysis because it may be correlated with the proportion of flight-capable individuals, the causal relationship between these variables was not clearly determined. We calculated mean pairwise distances between individuals within a population as an index of genetic diversity. Climatic condition variables included annual mean temperature, annual rainfall, and maximum snow depth at each sampling site based on meteorological data from 1971 to 2000 (Japan Meteorological Agency 2002). We performed a GLM analysis with logit link function using R 2.11.1. The backward stepwise method of model selection was conducted using the stepAIC function in the package MASS implemented in R 2.11.1 to select the best model. Only sites with five or more samples for flight muscle conditions and genetic data were included in the analysis.

## Results

### Genetic differentiation among geographic populations

**Figure 2:**
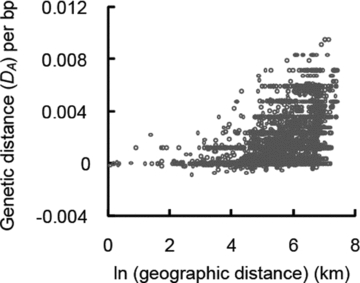
Relationship between geographic distance (ln-transformed, km) and Nei's distance *D_A_* per base pair.

A total of 396 sequences were obtained. For populations with five or more samples, the population means (±SD) of haplotype and nucleotide diversities were 0.66 ± 0.26 and 0.0020 ± 0.0014, respectively (Table S1). There was significant genetic differentiation among populations (Φ_ST_ = 0.39; *P* < 0.001), although genetic variances were higher within populations than among populations (within-population variance = 0.82, among-population variance = 0.53). The Mantel test showed a significant isolation-by-geographic distance pattern (Mantel's *r* = 0.476; *P* < 0.001; Fig. 2).

**Figure 3:**
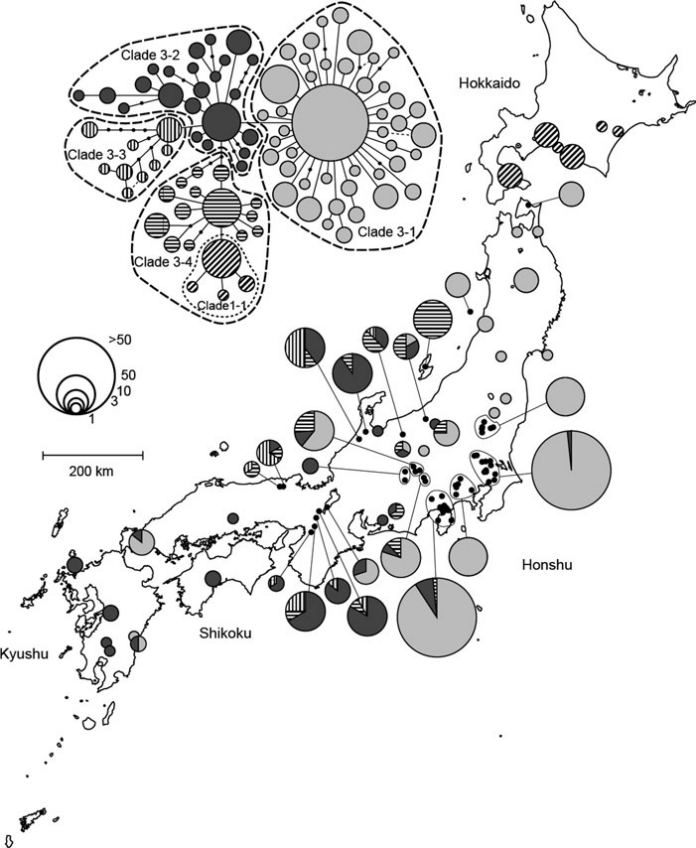
Haplotype network and nested clade design for the COI-II gene of *Necrophila japonica*, and the geographic distribution pattern of clades. Dots in the haplotype network represent missing haplotypes. Loops in networks have been resolved and alternative connections are shown by dashed lines. The geographic distributions of four 3-step clades and one 1-step clade at each sampling site are shown on the map.

Four three-step clades were determined in the statistical parsimony network of the mitochondrial haplotypes (Fig. 3). The geographic distribution pattern of haplotypes indicated that the populations of *N. japonica* were genetically differentiated among geographic regions. The largest clade was 3–1, whose haplotypes mainly occurred in northern and central Honshu. The haplotypes of clade 3–2 occurred in central and western Honshu, Shikoku, and Kyushu, while those of clade 3–3 were only found in central Honshu. The haplotypes of clade 3–4 mainly occurred in central Honshu, whereas clade 1–1, one of the one-step clades within clade 3–4, was found only in Hokkaido. The haplotypes from Hokkaido (clade 1–1) were closer to those from central Honshu than to those from northern Honshu (clade 3–1), which is adjacent to Hokkaido. All three-step clades exhibited nonsignificant SSD values, but clades 3–1, 3–2, and 3–4 had negative significant Fu's *Fs*, suggesting demographic expansion in these clades (Table 1).

**Table 1:** Number of samples with and without flight muscles and the tests of demographic expansion and population equilibrium for each clade of COI-II haplotypes. Nested clades are defined in Figure 3

	Flight muscle			Test of demographic expansion		Test of population equilibrium	
			
Clade	Present	Absent	Undetermined	SSD	*P*	Fu's *Fs*	*P*
3–1	147	52	41	0.000	0.930	−29.90	≤0.001
3–2	46	27	6	0.000	0.973	−17.72	≤0.001
3–3	13	3	1	0.012	0.596	−1.49	0.211
3–4	37	8	15	0.002	0.479	−11.52	≤0.001

### Distribution pattern of flight muscle dimorphisms

**Figure 4:**
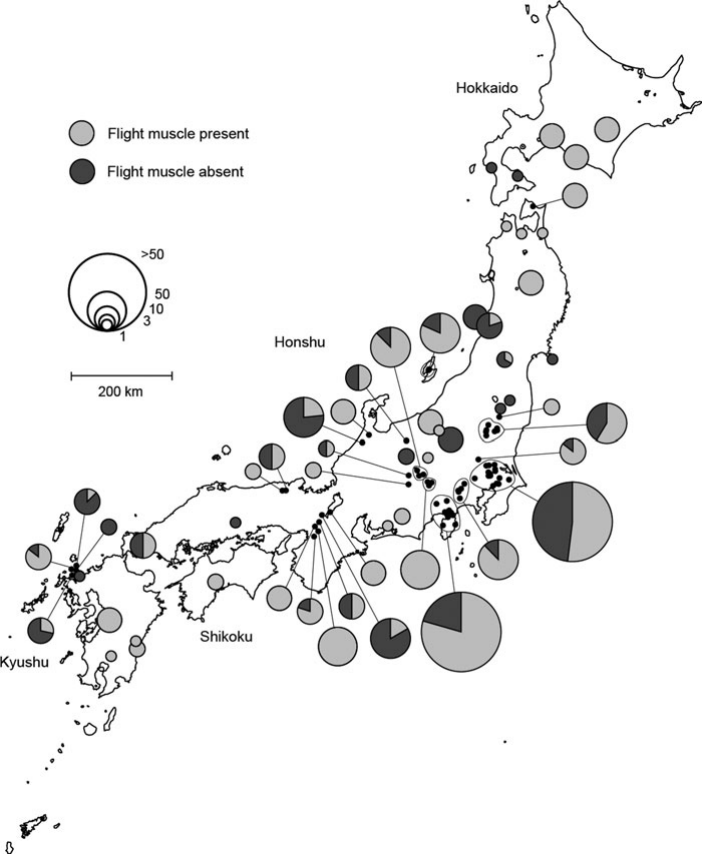
Geographic distribution pattern of flight muscle condition in *Necrophila japonica* in the Japanese archipelago.

We determined the flight muscle conditions for 242 female and 218 male beetles. The proportion of beetles with flight muscles was not different between sexes (66% vs. 71% for females and males, respectively; Fisher's exact test: *P* = 0.317). Dimorphic populations consisting of beetles with and without flight muscles were found throughout the Japanese archipelago (Fig. 4). However, beetles without flight muscles were absent from central Hokkaido, northern Honshu, and southern Kyushu.

**Figure 5:**
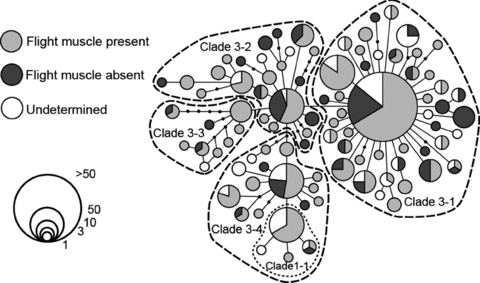
Haplotype network with flight muscle condition.

**Figure 6:**
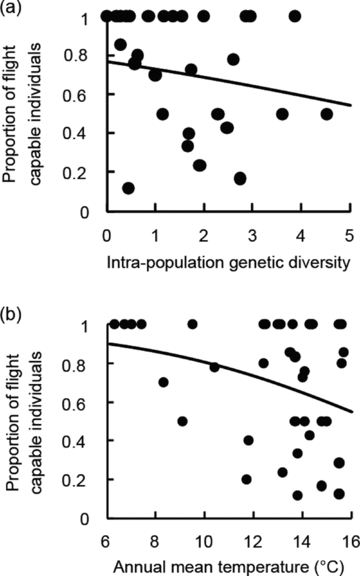
(a) Relationship between genetic diversity (average genetic distances between individuals) and the proportion of beetles with flight muscles in each population. Regression: *y* = exp(−0.043*x*+ 0.771)/{1 + exp(−0.043*x*+ 0.771)}. (b) Relationship between annual mean temperature and the proportion of beetles with flight muscles in each population. Regression: *y* = exp(−0.020*x*+ 3.467)/{1 + exp(−0.020*x*+ 3.467)}.

There was no significant association between flight muscle condition and the COI-II haplotype clade (Fisher's exact test: *P* = 0.112; Fig. 5; Table 1). GLM analysis determined that the best model for the proportion of beetles with flight muscles included two explanatory variables, population genetic diversity and annual mean temperature (Table S2). In the selected model, the proportion of muscled beetles was higher in populations with lower genetic diversity (*b* = –0.195, *P* = 0.017; Fig. 6a) and in populations at sites with lower annual mean temperatures (*b* = –0.017, *P* = 0.095; Fig. 6b).

## Discussion

We clarified the phylogeography and macroevolutionary pattern of flight-muscle dimorphism in *N. japonica*. The populations were not clearly clustered genetically by geographic regions in comparison to the cases of monomorphic wingless species (*Silpha longicornis*, *S. perforata*) of Silphinae in Japan (Ikeda et al. 2009). Although the strait between Honshu and Hokkaido has acted as a barrier to dispersal of wingless species, *N. japonica* has colonized Hokkaido. Given that demographic expansion was suggested for three-step clades of COI-II haplotypes with wide distribution ranges, recent gene flow and range expansion may have produced the observed phylogeographic pattern of *N. japonica*. Both flight-capable and flightless (i.e., lacking flight muscles) individuals occurred in most parts of the distribution area and in all clades of COI-II haplotypes. Thus, flight dimorphisms in *N. japonica*, which are likely maintained by inhabiting various habitats ranging from unstable river banks to relatively stable forests (Nagano and Suzuki 2003; Ikeda et al. 2008a, 2010; Shibuya et al. 2008), might enable rapid colonization of peripheral territories.

Flight-capable individuals rather than flightless (wingless or lacking flight muscles) individuals would more readily achieve colonization in peripheral or new habitats (e.g., den Boer 1970; Liebherr and Hajek 1986). Flight ability tends to be lost in long-lasting populations under stable habitat conditions (Harrison 1980; Roff 1990; Denno et al. 1991; Wagner and Liebherr 1992). For our GLM analysis of the proportion of flight-capable individuals, intrapopulation genetic diversity and habitat temperature were explanatory variables, although the effect of genetic diversity was only marginally significant. Populations newly established by flight-capable individuals may exhibit low genetic diversity due to bottlenecks, and habitats with low annual mean temperature (mainly at high altitudes or latitudes) may often correspond to newly colonized habitats. By contrast, populations in warmer regions may be more persistent and have retained larger population sizes due to weaker seasonal limitation, resulting in higher proportion of flightless individuals (Denno et al. 1991) and also in higher genetic diversity as was shown in a butterfly species (Orsini et al. 2008).

Given that there was little genetic divergence within Hokkaido populations and between northern and central Honshu populations, northern Honshu and central Hokkaido populations may have been established by expansion from central Honshu and southern Hokkaido populations, respectively, after the last glacial maximum. The Hokkaido population of *N. japonica* might have inhabited refugia in south Hokkaido during the last glacial maximum, as suggested for a flightless silphine species, *S. perforata* (Ikeda et al. 2009). The clade 3–4 haplotypes in Hokkaido are derived from those in central Honshu, but no haplotype of clade 3–4 was found in northern Honshu, the intermediate region between central Honshu and Hokkaido. In northern Honshu, populations with clade 3–4 haplotypes may have become extinct or these haplotypes may have been replaced by other haplotypes. The proportion of flight-capable individuals was also high in southern Kyushu, and demographic expansion was suggested in clade 3–2, which occurred in this region. Because the central haplotype of clade 3–2 mainly occurred in central Honshu, range expansion might have taken place in the populations of clade 3–2 from central Honshu to southern Kyushu.

Flight-muscle dimorphisms in insects may not always reveal a phylogeographic pattern consistent with that of *N. japonica*. *Phelotrupes laevistriatus*, a geotrupid beetle in Japan, possessed flight muscle dimorphisms similar to those of *N. japonica*, but exhibited distinct differentiations in both flight-muscle state and mitochondrial lineage with latitude in Japan (Ohta et al. 2009). Southern populations of *P. laevistriatus* were flight-capable, whereas northern (Hokkaido) populations were flightless, and dimorphic populations occurred in the intermediate zone. Populations in different regions possessed distinct COI lineages, which may have diverged during the Pliocene and Pleistocene (Ohta et al. 2009). In *P. laevistriatus*, flightlessness may be related to adaptation to environmental conditions that vary with latitude, but the adaptive significance of flightlessness remains unknown. For *Heptophylla picea* (Scarabaeidae), another beetle species exhibiting flight muscle dimorphism in Japan, dimorphic populations occur widely, but monomorphic flight-capable populations tend to exist in the northern-most and southern-most regions (Tada et al. 1995). Although population genetic data are not available for this species, the phylogeographic pattern may be similar to that of *N. japonica*.

To further elucidate the mechanism of flight loss in *N. japonica*, it is necessary to conduct additional studies such as microscale field surveys and breeding experiments to clarify the genetics behind flight-muscle dimorphisms and phylogeographic analysis using multiple nuclear loci to corroborate the results from COI-II gene sequences. Given that loss of flight appears to have occurred in diverse insect lineages, such studies will provide new insight into the mechanism of flight loss and related ecological evolution in insects, one of the major processes of insect evolution (Roff 1990).

## Supporting Information

**Table S1.** Coordinates of longitude and latitude, number of samples with and without flightmuscles, haplotype diversity, and nucleotide diversity in each site.

**Table S2.** Akaike's Information Criterion (AIC) values for the generalized linearmodels for the proportion of flight-capable individuals in response to four explanatory variables.
