# Supplementary material for: Macroscale evolutionary patterns of flight muscle dimorphism in the carrion beetle Necrophila japonica
Source: Ecol Evol. 2011 Sep;1(1):97–105. doi: 10.1002/ece3.15 (PMC3287380; doi:10.1002/ece3.15)
Supplement: Supplementary file 1 [file ece30001-0097-SD1.doc]

**Table S1** Coordinates of longitude and latitude, number of samples with and without flight muscles, haplotype diversity and nucleotide diversity in each site.

|  | Coordinates | |  | Flight muscle | | | |  | Genetic analysis | | |
| --- | --- | --- | --- | --- | --- | --- | --- | --- | --- | --- | --- |
| Locality | Latitude | Longitude |  |  |  |  | Undeter- |  |  | Haplotype | Nucleotide |
| number | (°N) | (°E) |  | *n* | Present | Absent | mined |  | *n* | diversity | diversity |
| 1 | 42.93 | 143.22 |  | 6 | 6 | 0 | 0 |  | 1 | — | — |
| 2 | 42.85 | 143.67 |  | 1 | 0 | 0 | 1 |  | 1 | — | — |
| 3 | 42.79 | 141.70 |  | 7 | 7 | 0 | 0 |  | 7 | 0.29 | 0.00034 |
| 4 | 42.57 | 141.94 |  | 1 | 0 | 0 | 1 |  | 1 | — | — |
| 5 | 42.37 | 142.30 |  | 7 | 7 | 0 | 0 |  | 6 | 0.00 | 0.00000 |
| 6 | 42.13 | 140.02 |  | 1 | 0 | 1 | 0 |  | 0 | — | — |
| 7 | 41.98 | 140.67 |  | 4 | 0 | 1 | 3 |  | 4 | 0.83 | 0.00139 |
| 8 | 41.40 | 141.15 |  | 4 | 4 | 0 | 0 |  | 4 | 0.00 | 0.00000 |
| 9 | 40.99 | 140.39 |  | 1 | 1 | 0 | 0 |  | 0 | — | — |
| 10 | 40.82 | 141.38 |  | 1 | 1 | 0 | 0 |  | 1 | — | — |
| 11 | 40.82 | 140.82 |  | 1 | 1 | 0 | 0 |  | 1 | — | — |
| 12 | 39.83 | 141.04 |  | 5 | 5 | 0 | 0 |  | 4 | 0.83 | 0.00178 |
| 13 | 39.19 | 139.55 |  | 4 | 0 | 4 | 0 |  | 4 | 0.00 | 0.00000 |
| 14 | 38.97 | 139.94 |  | 5 | 1 | 4 | 0 |  | 3 | 0.67 | 0.00079 |
| 15 | 38.29 | 141.57 |  | 1 | 0 | 1 | 0 |  | 1 | — | — |
| 16 | 38.26 | 140.36 |  | 3 | 1 | 2 | 0 |  | 1 | — | — |
| 17 | 38.06 | 138.29 |  | 1 | 1 | 0 | 0 |  | 0 | — | — |
| 18 | 38.05 | 138.37 |  | 12 | 8 | 2 | 2 |  | 12 | 0.44 | 0.00076 |
| 19 | 37.41 | 140.47 |  | 1 | 0 | 1 | 0 |  | 1 | — | — |
| 20 | 37.18 | 139.99 |  | 1 | 0 | 1 | 0 |  | 0 | — | — |
| 21 | 37.09 | 140.19 |  | 2 | 2 | 0 | 0 |  | 1 | — | — |
| 22 | 36.98 | 138.43 |  | 7 | 7 | 0 | 0 |  | 6 | 0.93 | 0.00341 |
| 23 | 36.93 | 139.93 |  | 3 | 1 | 2 | 0 |  | 3 | 1.00 | 0.00158 |
| 24 | 36.86 | 138.66 |  | 1 | 1 | 0 | 0 |  | 1 | — | — |
| 25 | 36.83 | 139.89 |  | 10 | 2 | 3 | 5 |  | 10 | 0.84 | 0.00201 |
| 26 | 36.82 | 140.12 |  | 1 | 1 | 0 | 0 |  | 0 | — | — |
| 27 | 36.81 | 140.14 |  | 3 | 2 | 1 | 0 |  | 2 | 0.00 | 0.00000 |
| 28 | 36.80 | 140.08 |  | 2 | 1 | 1 | 0 |  | 2 | 0.00 | 0.00000 |
| 29 | 36.79 | 139.85 |  | 2 | 1 | 0 | 1 |  | 2 | 1.00 | 0.00119 |
| 30 | 36.70 | 137.19 |  | 1 | 0 | 0 | 1 |  | 1 | — | — |
| 31 | 36.69 | 139.88 |  | 2 | 2 | 0 | 0 |  | 2 | 0.00 | 0.00000 |
| 32 | 36.69 | 136.88 |  | 17 | 4 | 13 | 0 |  | 10 | 0.76 | 0.00227 |
| 33 | 36.68 | 138.95 |  | 4 | 0 | 4 | 0 |  | 4 | 0.50 | 0.00238 |
| 34 | 36.63 | 137.84 |  | 8 | 4 | 4 | 0 |  | 8 | 0.96 | 0.00428 |
| 35 | 36.53 | 136.73 |  | 10 | 10 | 0 | 0 |  | 10 | 0.73 | 0.00161 |
| 36 | 36.35 | 137.84 |  | 3 | 0 | 3 | 0 |  | 3 | 1.00 | 0.00633 |
| 37 | 36.31 | 138.38 |  | 1 | 1 | 0 | 0 |  | 1 | — | — |
| 38 | 36.24 | 139.71 |  | 7 | 6 | 1 | 0 |  | 7 | 0.29 | 0.00034 |
| 39 | 36.23 | 139.69 |  | 1 | 0 | 0 | 1 |  | 1 | — | — |
| 40 | 36.11 | 140.07 |  | 6 | 5 | 1 | 0 |  | 3 | 0.67 | 0.00079 |
| 41 | 36.11 | 139.89 |  | 3 | 3 | 0 | 0 |  | 3 | 1.00 | 0.00158 |
| 42 | 36.10 | 139.95 |  | 4 | 1 | 3 | 0 |  | 4 | 1.00 | 0.00178 |
| 43 | 36.03 | 140.10 |  | 5 | 5 | 0 | 0 |  | 1 | — | — |
| 44 | 36.01 | 140.13 |  | 2 | 2 | 0 | 0 |  | 0 | — | — |
| 45 | 35.99 | 138.13 |  | 9 | 7 | 2 | 0 |  | 8 | 0.93 | 0.00310 |
| 46 | 35.93 | 139.94 |  | 1 | 0 | 1 | 0 |  | 0 | — | — |
| 47 | 35.93 | 139.96 |  | 6 | 2 | 4 | 0 |  | 6 | 0.80 | 0.00198 |
| 48 | 35.92 | 138.28 |  | 7 | 3 | 0 | 4 |  | 7 | 0.86 | 0.00396 |
| 49 | 35.91 | 139.96 |  | 17 | 2 | 15 | 0 |  | 9 | 0.22 | 0.00053 |
| 50 | 35.90 | 139.94 |  | 15 | 8 | 3 | 4 |  | 15 | 0.92 | 0.00206 |
| 51 | 35.89 | 138.17 |  | 8 | 4 | 0 | 4 |  | 8 | 0.86 | 0.00378 |
| 52 | 35.88 | 137.92 |  | 2 | 1 | 1 | 0 |  | 1 | — | — |
| 53 | 35.87 | 140.62 |  | 2 | 2 | 0 | 0 |  | 0 | — | — |
| 54 | 35.85 | 140.19 |  | 6 | 1 | 1 | 4 |  | 6 | 0.80 | 0.00119 |
| 55 | 35.77 | 138.39 |  | 7 | 7 | 0 | 0 |  | 7 | 0.48 | 0.00057 |
| 56 | 35.74 | 138.47 |  | 1 | 1 | 0 | 0 |  | 0 | — | — |
| 57 | 35.74 | 139.32 |  | 6 | 3 | 3 | 0 |  | 2 | 0.00 | 0.00000 |
| 58 | 35.74 | 139.54 |  | 1 | 0 | 1 | 0 |  | 0 | — | — |
| 59 | 35.73 | 140.17 |  | 5 | 5 | 0 | 0 |  | 5 | 0.90 | 0.00238 |
| 60 | 35.72 | 140.17 |  | 2 | 1 | 1 | 0 |  | 0 | — | — |
| 61 | 35.71 | 137.91 |  | 2 | 2 | 0 | 0 |  | 2 | 0.00 | 0.00000 |
| 62 | 35.69 | 138.43 |  | 18 | 17 | 0 | 1 |  | 18 | 0.63 | 0.00185 |
| 63 | 35.69 | 140.04 |  | 4 | 0 | 4 | 0 |  | 1 | — | — |
| 64 | 35.62 | 139.24 |  | 1 | 1 | 0 | 0 |  | 1 | — | — |
| 65 | 35.60 | 139.21 |  | 7 | 5 | 0 | 2 |  | 7 | 0.71 | 0.00102 |
| 66 | 35.59 | 139.21 |  | 12 | 12 | 0 | 0 |  | 12 | 0.58 | 0.00139 |
| 67 | 35.52 | 139.60 |  | 3 | 0 | 0 | 3 |  | 3 | 0.67 | 0.00158 |
| 68 | 35.49 | 134.84 |  | 6 | 3 | 3 | 0 |  | 6 | 0.80 | 0.00538 |
| 69 | 35.48 | 134.80 |  | 3 | 3 | 0 | 0 |  | 3 | 0.67 | 0.00475 |
| 70 | 35.43 | 139.22 |  | 1 | 1 | 0 | 0 |  | 1 | — | — |
| 71 | 35.39 | 138.86 |  | 10 | 7 | 3 | 0 |  | 10 | 0.53 | 0.00119 |
| 72 | 35.32 | 138.61 |  | 3 | 3 | 0 | 0 |  | 3 | 0.67 | 0.00238 |
| 73 | 35.15 | 138.84 |  | 5 | 5 | 0 | 0 |  | 5 | 0.40 | 0.00048 |
| 74 | 35.15 | 138.93 |  | 7 | 3 | 4 | 0 |  | 7 | 0.71 | 0.00294 |
| 75 | 35.14 | 138.94 |  | 5 | 3 | 1 | 1 |  | 5 | 0.40 | 0.00095 |
| 76 | 35.13 | 138.93 |  | 1 | 1 | 0 | 0 |  | 1 | — | — |
| 77 | 35.12 | 138.99 |  | 1 | 1 | 0 | 0 |  | 1 | — | — |
| 78 | 35.10 | 135.99 |  | 7 | 7 | 0 | 0 |  | 7 | 0.86 | 0.00351 |
| 79 | 35.08 | 137.68 |  | 3 | 2 | 0 | 1 |  | 3 | 1.00 | 0.00317 |
| 80 | 35.07 | 138.90 |  | 1 | 1 | 0 | 0 |  | 1 | — | — |
| 81 | 35.05 | 138.90 |  | 2 | 2 | 0 | 0 |  | 2 | 1.00 | 0.00119 |
| 82 | 35.03 | 135.79 |  | 12 | 2 | 10 | 0 |  | 12 | 0.83 | 0.00326 |
| 83 | 34.90 | 139.10 |  | 29 | 22 | 7 | 0 |  | 21 | 0.50 | 0.00068 |
| 84 | 34.89 | 135.69 |  | 10 | 5 | 5 | 0 |  | 7 | 0.86 | 0.00272 |
| 85 | 34.89 | 138.77 |  | 10 | 10 | 0 | 0 |  | 10 | 0.20 | 0.00024 |
| 86 | 34.86 | 137.40 |  | 1 | 1 | 0 | 0 |  | 1 | — | — |
| 87 | 34.79 | 133.65 |  | 1 | 0 | 1 | 0 |  | 1 | — | — |
| 88 | 34.76 | 135.67 |  | 4 | 4 | 0 | 0 |  | 0 | — | — |
| 89 | 34.70 | 135.69 |  | 12 | 11 | 0 | 1 |  | 12 | 0.94 | 0.00461 |
| 90 | 34.57 | 135.63 |  | 5 | 4 | 1 | 0 |  | 3 | 1.00 | 0.00633 |
| 91 | 34.15 | 131.46 |  | 10 | 5 | 5 | 0 |  | 7 | 0.71 | 0.00136 |
| 92 | 33.60 | 129.86 |  | 8 | 1 | 7 | 0 |  | 0 | — | — |
| 93 | 33.58 | 129.76 |  | 7 | 6 | 1 | 0 |  | 0 | — | — |
| 94 | 33.56 | 129.88 |  | 3 | 0 | 3 | 0 |  | 3 | 0.67 | 0.00238 |
| 95 | 33.53 | 133.25 |  | 2 | 2 | 0 | 0 |  | 2 | 1.00 | 0.00238 |
| 96 | 33.42 | 129.91 |  | 1 | 0 | 1 | 0 |  | 0 | — | — |
| 97 | 33.42 | 129.73 |  | 7 | 2 | 5 | 0 |  | 0 | — | — |
| 98 | 32.64 | 130.82 |  | 5 | 5 | 0 | 0 |  | 2 | 1.00 | 0.00119 |
| 99 | 32.15 | 131.50 |  | 1 | 1 | 0 | 0 |  | 1 | — | — |
| 100 | 32.11 | 131.52 |  | 2 | 2 | 0 | 0 |  | 2 | 1.00 | 0.00238 |
| 101 | 32.04 | 130.82 |  | 1 | 0 | 0 | 1 |  | 1 | — | — |
| 102 | 31.89 | 130.90 |  | 1 | 1 | 0 | 0 |  | 1 | — | — |

**Table S2** Akaike’s Information Criterion (AIC) values for the generalized linear models for the proportion of flight-capable individuals in response to four explanatory variables. The worst-performing environmental parameter was excluded from the model in each step. The best model in each step is shown in bold.

| Factor | AIC |
| --- | --- |
| First step |  |
| Starting model (+Gene+Temp+Rain+Snow) | 345.16 |
| **-Snow** | **343.16** |
| -Rain | 343.27 |
| -Gene | 345.44 |
| -Temp | 346.48 |
|  |  |
| Second step |  |
| Starting model (+Gene+Temp+Rain) | 343.16 |
| **-Rain** | **341.29** |
| -Gene | 344.06 |
| -Temp | 347.46 |
|  |  |
| Third step |  |
| **Starting model (+Gene+Temp)** | **341.29** |
| -Gene | 342.06 |
| -Temp | 345.79 |

Gene: genetic diversity; Temp: annual mean temperature; Rain: annual rainfall; Snow: maximum snow depth.
